# Effectiveness of Cognitive-Behavioral Therapy Modified for Inpatients with Depression

**DOI:** 10.5402/2012/461265

**Published:** 2011-12-06

**Authors:** Andrew C. Page, Geoff R. Hooke

**Affiliations:** ^1^The University of Western Australia, Crawley, WA 6009, Australia; ^2^Perth Clinic, West Perth, WA 6005, Australia

## Abstract

The effectiveness among inpatients with depression of a modified cognitive behavior therapy (CBT) program was examined. A group of 300 inpatient admissions with a primary diagnosis of depression attending a private psychiatric clinic were assessed at the beginning and end of a two-week CBT program. The effectiveness of the treatment was demonstrated by improvements on the Beck depression inventory (BDI), the health of the nation outcome scales, locus of control of behaviour scale, and the global assessment of function. The changes on the BDI for patients with depression were benchmarked against estimates generated from published studies. The degree of change in a two-week period for inpatients with depression was similar to that observed in efficacy studies of CBT that typically run over a more extended time. Implications for integrating CBT with inpatient services are discussed.

## 1. Introduction

Translating empirically validated treatments into routine clinical practice is an essential activity that requires a multitude of factors to be addressed. For any given disorder, some of these factors include the type of patients being treated, the setting in which treatment is delivered (including the funding arrangements and costs incurred), the attitudes and perceptions of staff about the treatment, and the other psychological and physical disorders, diseases, and disabilities that may co-occur with the condition being treated [1]. These various factors mean that the translation will first involve a thoughtful adaptation to the given setting that takes into account local issues, current practices, staff attitudes, and funding arrangements. Secondly, translation will involve evaluation of the effectiveness of the resulting treatment plus an empirical consideration of factors believed to influence outcome so that treatment can be further improved [2, 3]. The present paper reports an attempt to translate a cognitive-behavioral program for depression to an inpatient hospital setting.

There is sound evidence supporting cognitive-behavioral therapy (CBT) in the treatment of depression [4], but little direction about adaptation to inpatient settings. When considering translation into an inpatient setting, a number of considerations are important. First, people with depression who require inpatient admissions often do so at a time of crisis or extreme symptom severity, and the aim is to quickly and safely return them to levels of independent function. For instance, inpatient treatment may be appropriate if there is a risk of suicide, but a goal will be to reduce symptoms rapidly, so the need for a restricted environment is removed swiftly. Within these constraints, the typical 20-week format of CBT for depression is not necessarily the most suitable. In addition, individuals with depression who do not require hospitalization may find an optimal treatment format to be brief, weekly sessions. However, for individuals who are in a hospital, the logistics of organizing a more time-intensive program are less (especially since the patients might otherwise not be occupied for much of the day), and hospital staff are already present. Thus, an intensive day-long format of CBT becomes not only a viable option, but also a potentially good use of time. Third, given the constant turnover within a hospital, it is harder to mount disorder-specific treatments because the patients will possess various degrees of comorbidity and might not fit neatly into diagnostic categories, yet they may benefit from a CBT program. For reasons such as these, Perth Clinic developed an intensive CBT program suitable for inpatients [5]. It is a closed-group program that is open to various diagnostic groups (but mainly depression and anxiety) during office hours for a two-week period. The first question to answer regarded the degree to which the outcomes reported in the published literature regarding group CBT for depression would generalize to this different format.

The second focus of the present study concerned variables related to outcome. In a hospital setting, many factors are uncontrollable. For instance, the degree and type of comorbidity is a potentially important moderator of treatment success. In addition, success may be moderated by the frequency of depression (i.e., whether the current presentation is a single or a recurrent episode) or the severity of the depression. According to Beutler [2], “prognosis is attenuated by patient complexity/chronicity, and by an absence of patient distress. Facilitating social support enhances the likelihood of good outcome among patients with complex/chronic problems.” Therefore, to assist with future treatment planning, it was necessary to consider the degree to which patient-related variables affected outcome.

In conducting an evaluation of the current program, there were a variety of constraints imposed by the context. Chiefly, it was not possible to randomly assign patients to a control condition, and therefore, a variety of methods were used to evaluate the size of the treatment effect and to address some alternative explanations of a pre/posttreatment change. To increase confidence in the presumption that any pre/postchanges were associated with the provision of treatment, outcomes were compared against inpatients who did not receive the CBT program and those who began the treatment but did not complete. The former provide an indication of the extent of improvement without the program, but due to nonrandom assignment to conditions, it is possible that patients with poorer outcomes were not encouraged to enroll in the CBT program. This problem is partly addressed by examining the patients who enrolled in but did not complete the CBT program. Ideally, these patient groups will be similar at admission to hospital, but patients who complete the CBT program will have superior outcomes. Another way to address the causal role played by the CBT program is to examine the specificity of treatment changes. Since CBT is aimed at changing emotions, mood-related symptoms should change more during the CBT program than at other points in the inpatient admission. Finally, to evaluate the size of the treatment change, published treatment data were used to generate benchmark pre- and posttreatment data.

One study that could serve as a benchmark for patients with depression was reported by Peterson and Halstead [6]. They examined the effectiveness of group CBT for depressed (i.e., major depressive disorder single-episode and recurrent, dysthymic disorder, depressive disorder not otherwise specified, and adjustment disorder with depressed mood) outpatients. Their study found a reduction from a pretreatment mean of 23.1 on the Beck depression inventory (BDI) [7] to 14.4 by posttreatment. Another relevant benchmark can be derived from studies that have conducted group psychotherapy to treat depression and used the BDI as an outcome measure. McDermut et al. [8] reviewed these studies found 35 such studies from which they calculated an overall pretreatment mean on the BDI of 23.9 and a posttreatment mean of 12.3. This review incorporated a variety of treatments; these values represent an acceptable benchmark because most of the studies included were versions of CBT. Excluding those studies that were not CBT, the values improved the estimate of the treatment effect somewhat (BDI before treatment of 25.5 to 11.6 at after treatment), for clients with a mean age of 36 and a mean number of 20 therapy hours. A final benchmark was an outpatient evaluation of group CBT for depressed patients treated within diagnostically heterogenous groups [9]. Their outcomes for the BDI revealed a decline from 26.9  (SD = 8.9) to 16.5  (SD = 11.3). The effect size (Cohen's d) of this change was very large (i.e., 1.2) with 59% of patients demonstrating a clinically significant change after treatment.

Thus, the aim of the present study was to examine the effectiveness for inpatients with depression treated with a CBT program modified to run intensively over a two-week period. 

## 2. Method

### 2.1. Patient Sample

The total patient population comprised an archival dataset of 998 consecutive inpatient admissions diagnosed with a major depressive disorder from July 1996 to March 1999, who spent at least one day in hospital and who completed a BDI at admission to hospital. Patients were diagnosed using a clinical interview according to DSM-IV by their treating psychiatrist. Each psychiatrist continued to provide ongoing management for their patients. The decision to admit the person as an inpatient was made by the psychiatrist and was made when it was apparent that the required treatment could not be provided optimally while the patient was in the community. For instance, greater nursing care may be required to manage suicide risk. The total patient group had a mean age of 43.2  (SD = 14.4), and 73% were female. Of the total sample, 46% were married, 26% were separated or divorced, 17% were widowed, and the remainder were never married. The depressive disorders were classified as a single episode among 52% of cases and recurrent among the remaining 48%; the depression was the primary diagnosis among 82% of patients and secondary to another diagnosis among the remainder. The mean BDI score at admission was 31.3  (SD = 11.7). For those with a primary diagnosis of depression, the most common recorded comorbid disorders were anxiety disorders (18%), substance use (12%), and personality disorders (5%), and 58% of patients had at least one medical condition coded on Axis III. Patients spent an average of 12.3 days in hospital (SD = 8.9; range of 2–66 days). Of the total patient group, 225 began the CBT program during their inpatient stay or immediately upon discharge, and 201 (89%) completed the program. Patients had provided consent for their data to be used for research and evaluation purposes, and the University's Human Research and Ethics Committee approved the analysis of the deidentified data.

### 2.2. Description of Treatment

People admitted as inpatients were expected to participate in available treatment options. In addition to medications managed by their treating psychiatrist and the CBT program, a variety of problem-focused open group inpatient programs are available, including an acute care program, a substance abuse program, and an interpersonal therapy program. However, the present study focused on patients' outcomes during a CBT program. Thus, while patients may have had exposure to other treatments before or after the CBT program, they were not engaged in any other treatment (except concurrent psychopharmacotherapy) at the time. The type of pharmacotherapy at admission, during the inpatient stay or at discharge, was not collected, but almost all inpatients would be on one type of medication.

Perth Clinic's CBT program is a closed group for up to eight patients, conducted over a period of ten working days [5, 10]. Each day consists of four 90-minute sessions. The program begins with problem identification and goal setting, leading to psychoeducation and cognitive therapy. The cognitive therapy draws upon the work of both Beck [7] and Ellis and Harper [11] and involves self-monitoring and the identification and challenging of irrational beliefs. Behavioral interventions such as anxiety management and stress reduction are taught (e.g., relaxation, breathing control, etc.), and patients engage in pleasant events scheduling and behavioral assignments to practise newly developed skills. In the latter part of treatment, the focus shifts to self-esteem, assertion, and communication training and concludes with a relapse prevention module. Each patient is supplied with a standard manual that covers these components. The structure allows sufficient time and flexibility for the discussion of both group and individual issues. There is also a supporters' session, where each participant is invited to attend with a supporter. The aim of this session is to increase awareness of psychological disorders, the nature of treatment, and helpful caring behaviors. Patients could complete the CBT program as an inpatient, as a daypatient (immediately following discharge), or being discharged from inpatient status at some point within the program [12]. The average time between admission and initiation of the CBT program was 4.9 days (SD = 7.4).

### 2.3. Design

All patients received the questionnaires at admission, discharge, and where applicable, at the beginning and end of the CBT program. Questionnaires were sent to all patients who completed the CBT program at a six-week followup. Staff-rated measures were administered at admission and discharge by ward staff and at the beginning and end of the CBT program by the treating therapists (clinical psychologists and occupational therapists).

### 2.4. Measures

Patients completed the Beck depression inventory [13]. The BDI is a 21-item self-report scale designed to measure the level of depression among clinical and non clinical populations and is widely used in research on depression. A high score reflects elevated depression. In addition, patients completed the locus of control of behaviour scale (LCB) [14] to assess their sense of control over themselves and their lives. A low score-indicates an internal sense of control. The Rosenberg self-esteem scale (RSES) [15] was administered to measure general self-concept and consists of ten items using a four-point Likert-type response format. A high score reflects elevated self-esteem.

Clinic staff rated patients' general level of psychiatric symptoms using the health of a nation outcome scales (HoNOS) [16, 17] and global assessment of function (GAF) [18]. The HoNOS is a 12-item clinician-rated scale that is comprehensive in coverage, clinically relevant, and quick to administer. HoNOS was introduced to the clinic in the middle of 1997, and therefore data are not available for one year of the study.

## 3. Results

### 3.1. Effectiveness of Treatment

The effectiveness of the CBT was evaluated in a variety of ways. First, the change in symptoms from admission, pre-CBT, post-CBT, to 6-week followup was examined. The BDI scores improved somewhat from admission (*M* = 30.3) to pre-CBT (*M* = 24.7), *F*(1,152) = 60.76, *η* ^2^ = .29, *P* < .001, markedly from pre- to post-CBT (*M*  12.2), *F*(1,152) = 299.90, *η* ^2^ = .66, *P* < .001, and remained stable until followup (*M* = 12.2), *F*(1,152) = 0.003, *η* ^2^ = .00, *P* = ns.

To compare the effectiveness of the present form of therapy with the results typically obtained in research trials, a 95% confidence interval was placed around the difference between pre- and posttreatment BDI scores and compared against the benchmark values identified in the Introduction. Thus, the 95% confidence interval around the difference of 12.5 between the pre- and posttreatment BDI scores extended from 11.1 to 13.9. The mean difference of 11.6 for studies of group psychotherapy [8] for depression was within this confidence interval (as was the 13.9 difference if these studies were limited to those using CBT), supporting that the conclusion that the difference obtained in the present investigation was comparable to that found with group treatment for depression generally. Another way to examine the overall effectiveness was to examine the clinical significance of the outcomes. Calculating a reliable change index [19] and using the normative data on the BDI described by Robinson et al. [20], 60.2% of patients demonstrated clinically significant improvement [19] after treatment.

Comparing the present results with those of in the depressed outpatient sample [9], it is apparent that inpatients were more severe at admission than the outpatient population (i.e., *M* = 30.3 versus 26.9), but their scores had declined to less severe (i.e., *M* = 24.7) by the start of the CBT program. This is consistent with a treatment model within which acutely unwell patients are first stabilised and then gains consolidated in a CBT program. The number of patients who demonstrated a clinically significant improvement from pre- to post-CBT (i.e., 60.2%) was similar to that found in an outpatient setting (i.e., 59%).

These changes in outcome were also observable on other clinician-rated and self-reported measures. In terms of the staff-rated GAF, the scores rose from admission (*M* = 49.3) to pre-CBT (*M* = 56.9), *F*(1,120) = 32.00, *η* ^2^ = .21, *P* < .001, and continued to increase from pre- to post-CBT (*M* = 69.1), *F*(1,120) = 219.65, *η* ^2^ = .65, *P* < .001. Patients' LCB scores did not increase from admission (*M* = 47.9) to pre-CBT (*M* = 47.2), *F*(1,151) = 1.24, *η* ^2^ = .01, *P* = ns, became substantially more by post-CBT (*M* = 38.2), *F*(1,151) = 144.14, *η* ^2^ = .49, *P* < .001, but it became marginally more external again by the 6-week followup (*M* = 40.0), *F*(1,151) = 6.51, *η* ^2^ = .04, *P* < .05. The patients' self-esteem scores did not improve from admission (*M* = 23.5) to pre-CBT (*M* = 23.6), *F*(1,102) = 0.03, *η* ^2^ = .000, *P* = ns, but they became more internal from pre to post-CBT (*M* = 28.7), *F*(1,102) = 106.87, *η* ^2^ = .51, *P* < .001, and remained stable at the 6-week followup (*M* = 28.9), *F*(1,102) = 0.22, *η* ^2^ = .002, *P* = ns.

Although the changes during the CBT program are comparable to those in published studies, one possible interpretation of the present findings is that the patients would have improved to the same degree due to their hospital stay. To partly address this concern, the patients who completed the CBT program were compared at admission and discharge to hospital with those who did not begin the CBT program on the one hand and those who withdrew from the program on the other. There were significant differences between the groups across these two time intervals, *F*(2,602) = 3.74, *η* ^2^ = .01, *P* < .05. Follow-up tests revealed no significant differences between groups at admission, but the patients who had completed CBT were less depressed (*M* = 13.1) than those who never began CBT (*M* = 16.6), *t*(611) = 2.56, *P* < .05, who in turn were not different from those who had dropped out of CBT (*M* = 16.9), *t*(516) = 0.10, *P* = ns. Thus, despite the absence of differences at admission, those who completed the CBT program were the least depressed at discharge.

**Figure 1:**
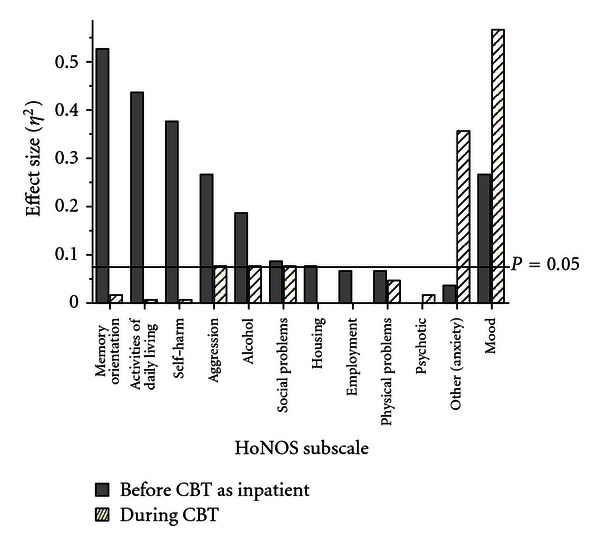
Mean effect size *η* ^2^ of change in the twelve HoNOS subscales from hospital admission to pre-CBT and from pre- to post-CBT.

To address the concern that the observed improvement may be due to the general effects of hospitalization rather than the specific effects of CBT, the profile of symptom change from admission to pre-CBT and then from pre- to post-CBT was examined. Assuming a degree of symptom specificity of treatments, because CBT is focussed on symptoms of depression and anxiety, the changes in these domains should be greater during CBT, whereas the earlier phase in an inpatient admission will be focussed on acute symptom management. To examine these issues, the changes from admission to pre-CBT and then to post-CBT in individual HoNOS items were examined. The data are summarized by presenting an effect size measure (*η* ^2^). What is apparent from Figure 1 is that the inpatient stay prior to entry into CBT is associated with small changes in anxiety and depression, but substantial improvements in memory/orientation, activities of daily living, self-harm, aggression, and substance problems, whereas the CBT program is associated with the greatest changes in emotional symptoms. Thus, the symptoms targeted by CBT change following the treatment, but domains that are not the focus of treatment shift little during the CBT program.

### 3.2. Influence of Clinical Variables on Outcome

Given the data showing that symptoms of depression improved during the time patients were in the CBT program, the effect on outcome of various clinical variables was examined. Of the variables examined there was no difference in BDI scores between patients from pre- to post-CBT for whom the depression was a single episode or a recurrent episode, *F*(1,146) = 2.37, *η* ^2^ = .02, *P* = ns, or for whom the depression was judged to be primary or secondary to another disorder, *F*(1,151) = 0.15, *η* ^2^ = .001, *P* = ns. Considering those patients for whom depression was the primary disorder, there was a hint that the total number of secondary diagnoses might be associated with a poorer outcome, *F*(1,97) = 2.98, *η* ^2^ = .03, *P* = .09, but there was no indication of poorer outcomes for patients with personality disorders, *F*(1,151) = 0.19, *η* ^2^ = .001, *P* = ns, affective disorders, *F*(1,151) = 0.15, *η* ^2^ = .001, *P* = ns, and neurotic disorders,  *F*(1,151) = 0.83, *η* ^2^ = .01, *P* = ns. Although there was no difference in BDI scores before and after CBT between patients with more secondary medical conditions, *F*(6,146) = 1.67, *η* ^2^ = .06, *P* = ns, there was a hint that patients with more secondary substance use problems might have worse outcomes, *F*(3,149) = 2.44, *η* ^2^ = .05, *P* = .07. Thus, there was no strong evidence in the present sample that presentations of major depression that were complicated by other Axis I, II, or III were any more resistant to a CBT program than the less complicated presentations.

**Figure 2:**
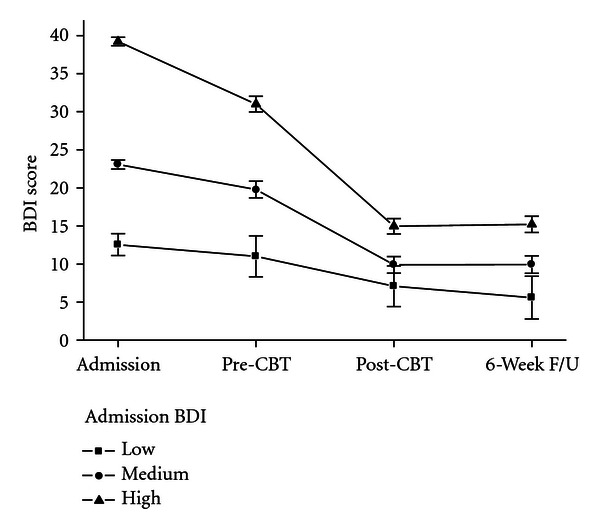
Changes in Beck depression inventory scores from hospital admission, pre-CBT, post-CBT, and six-week followup were broken down according to level of depression at admission.

However, there was one clinical variable that was strongly related to outcome and that was severity of depression at admission. Using a trecile split to divide patients into three equal-sized groups based on admission BDI scores, it was apparent that the change from admission to pre-CBT, *F*(2,150) = 7.20, *η* ^2^ = .09, *P* < .01, and from pre- to post-CBT, *F*(2,150) = 16.73, *η* ^2^ = .18, *P* < .001, was systematically related to severity. From Figure 2, it is apparent that the greatest changes were observed in those with the most severe problems, but that once CBT was finished, patients maintained the level of symptoms after treatment for the next six weeks, *F*(2,150) = 0.31, *η* ^2^ = .004, *P* = ns.

## 4. Discussion

The present study examined the effectiveness for patients with depression of a CBT program adapted to an inpatient psychiatric clinic. The intensive program brought about reductions in depression ratings compared to those found in the literature more generally, but importantly, it was able to achieve these gains within a two-week period, rather than the more usual 16–20 weeks of treatment. The speed of the treatment gains is important because patients who require inpatient treatment are often distressed and may represent a threat to their own safety. Therefore, a relatively rapid treatment means that a safer environment can be provided, but the period of time when they are removed from their typical routines and support is minimized. Thus, CBT for depression can be adapted to an intensive program suitable for delivery within the model of care found in inpatient settings. 

However, within inpatient settings, multiple interventions co-occur. For the present study, this means that it is not possible to use the data to conclude that the CBT program caused the symptom changes. However, this is an empirical issue that has already been answered numerous times in controlled efficacy studies [4], and the present question is related to the effectiveness of CBT in a particular context. In particular, it is also clear that packaging a CBT program as part of an inpatient treatment program can deliver comparable treatment outcomes to spaced treatments, but it did not achieve these outcomes at the expense of longevity of treatment gains. The gains observed were stable over the six-week followup, and there was no evidence that the more severe patients were any more likely to return to problematic levels following the termination of the program.

From a clinical perspective, a common issue raised among practitioners is that the treatments developed and then demonstrated in efficacy studies will not generalize to “real-world” clinical settings. In our experience, common reasons given for the lack of generalization include the greater severity of patients outside efficacy studies, the higher occurrence of comorbidity (especially substance use and personality disorders) since these patients can rarely be prohibited from receiving treatment in the way that they may be appropriately excluded from an efficacy study, and the setting itself. Therefore, it was curious to see that in the present sample, the only variable that was associated with differential outcomes was symptom severity such that the greatest change was observed in the patients who were most depressed.

Given that other studies have found that comorbidity and type of depression have an impact on outcome, it is intriguing that the present study failed to find these differences. One possible reason for the lack of a difference is the intensity of the program. There may be greater opportunity for the comorbid conditions to interfere with treatment when an intervention extends to months, whereas in an intense CBT program that fills most of the day, there may be less opportunity for these associated conditions to manifest themselves. For instance, a person with a substance use problem may be able to maintain harm-free use or abstinence for a couple of weeks while in treatment, whereas over a few months, this might be more difficult. Without random assignment to intensive or spaced treatment sessions, it is not possible to offer any more than this as a speculative suggestion, but the present data would encourage investigation of the benefits and costs of alternative forms of delivery.

In considering the results, there are a number of limitations that need to be borne in mind. First, the hospital is a private clinic where the patients are insured, and therefore, the extent of generalization to the public sector is not clear. It is possible that even though comorbid conditions are present, the severity or types of comorbidity that may be present in public hospitals (e.g., a greater frequency of psychotic disorders [10]) may mean that particular presentations were not represented in the current sample. Second, by its nature, the present investigation was a retrospective examination of effectiveness and not a randomized controlled trial. Therefore, there are a variety of issues that make it hard to conclude that the CBT program caused the observed effects. It is possible that the symptom changes that are observed during the CBT program may not be attributable to the program, but due to patient selection and other factors associated with hospital care (e.g., concurrent pharmacotherapy). While these cannot be ruled out, attempts were made to examine the degree to which these variables may have affected the data. The difference between inpatients who began the CBT program and those who did not suggests that the CBT program is providing benefits over and above “treatment as usual.” The benefits by discharge for people who began CBT and finished it, relative to those who dropped out, address the issue that staff selected CBT patients who were likely to improve regardless of the content of treatment, because those who dropped out were not different at admission or discharge from those who never began CBT. In addition, the apparent specificity of the treatment gains that occurred during CBT speaks against arguments about the general effect of being in hospital as causing the reductions in symptoms. However, while it remains possible that the treatment did not cause the observed outcomes, given that CBT has been demonstrated to be an efficacious treatment for depression and the current treatment effects were comparable to those observed in the efficacy studies, it is not unreasonable to presume that the application of an empirically validated treatment, albeit in a modified form, was largely responsible for the observed improvements. Third, the present study did not set out to establish the efficacy of CBT, but to examine the effectiveness within a clinical setting and relied on the patients' self-reports and the judgments of the treating clinicians. Although the internal validity of the ratings is less than those carried out by a person blind to the treatment, the external validity of the ratings (when conducted by clinicians who have spent two weeks with the patient) is greater.

Nonetheless, there is a possibility for bias as patients may have tried to please therapists, and the staff may have overestimated the change to a greater degree in the CBT program than in the hospital more generally. Finally, it is important to note that in contrast to efficacy studies, in a hospital context, it is not possible to exclude patients from a treatment with a BDI below a cut-off (e.g., [17]), that is, the decision to treat a particular patient will involve a consideration of the self-reported severity of the depression, but other factors will also be taken into account, and therefore, the sample contains individuals who might not meet the criteria for inclusion in an efficacy study, applying such a criterion (by excluding them from the analyses) if anything strengthens the conclusions of the present study, since the pre- to post-CBT scores change from 28.4 to 13.2; increasing the absolute treatment difference from 12.5 BDI points when all patients are included to 15.2 points.

Despite these limitations, it appears reasonable to conclude that CBT can be adapted to an inpatient psychiatric clinic, and the effectiveness of such a program is comparable to that observed in more homogenous patient samples found in efficacy studies. However, it is not clear from the present study the degree to which the apparent lack of comorbidity and type of depression to interfere with treatment outcomes is associated with the intensity of the treatment, the setting of the therapy, and the temporal location in the overall treatment program when CBT was delivered. Future research could investigate these questions and in so doing assist in clarifying the most effective way to deliver treatment in different settings to different patient presentations.

Another future research question could relate to the ability to deliver CBT to homogeneous patient groups. The patients in the present study were all diagnosed with depression, but they were treated within a group of patients where not all suffered from depression. The clinic provides a CBT program and makes it available to patients who might benefit from the program, and therefore a mixture of other primary diagnoses. Although it was not possible to evaluate the outcomes of these other patients (because the numbers of each diagnostic group were small, the outcome measures were not specific to the conditions, and there was not a sufficient literature on the disorders with the measures used to benchmark against the present data), it is clear that at least for people with depression, they can be treated alongside patients with other conditions without degradation of their treatment outcomes. The therapy staff observes that the mixed patient groups, rather than being a hindrance, are an assistance to therapy, since patients can assist one another in helping each other with their different problems. The degree to which this clinical observation can be replicated in a controlled study, it should be possible to start to isolate the factors that are beneficial about treating heterogenous patient groups.

The present study cannot identify the extent to which incorporating CBT into an inpatient program can reduce the overall length of hospital stay. The length of stay at the present clinic is relatively short, when compared to other private psychiatric clinics in Australia which treat similar patient groups (Moira Munro, personal communication, 27, October 2011), but different to public psychiatric hospitals (which tend to treat proportionally more people with schizophrenia). One possible explanation is the reliance on adjunctive psychotherapy, and we have shown elsewhere that the addition of psychotherapy enhances the outcomes following an inpatient stay [21]. However, the duration of hospital stay varies markedly across health care systems and countries, and therefore, this question is probably better addressed using multilevel modelling, where hospitals are nested with relevant units (e.g., healthcare system, nation, etc.).

In conclusion, the present study found that an evidence-based treatment for depression could be modified to an inpatient treatment setting without substantial loss of effectiveness. Clinically, this is important because people are often admitted as inpatients when suicide risk is high, and therefore, data consistent with the view that adding CBT to the treatment is available at such times is associated with a rapid reduction in symptoms. For a suicidal patient, a reduction in symptoms is going to be associated with a lowered risk of harm, and therefore, the sooner the reduction can be achieved, the sooner a less intensive treatment regime can the implemented. In addition, some concerns in generalizing from efficacy studies to clinical effectiveness that arise due to complexity of patients (e.g., comorbidity) were addressed, and no strong evidence was found that these issues compromised outcomes in the present study.
